# Effectiveness of DArTseq markers application in genetic diversity and population structure of indigenous chickens in Eastern Province of Rwanda

**DOI:** 10.1186/s12864-024-10089-5

**Published:** 2024-02-19

**Authors:** Valentin Mujyambere, Kwaku Adomako, Oscar Simon Olympio

**Affiliations:** 1 Department of Animal Production, School of Veterinary Medicine, University of Rwanda, Nyagatare, Rwanda; 2 Department of Animal Science, Faculty of Agriculture, Kwame Nkrumah University of Science and Technology, Kumasi, Ghana; 3 Department of Animal Production, University of Rwanda (UR), P.O. Box 57, Nyagatare, Rwanda; 4 Department of Animal Science, Kwame Nkrumah University of Science and Technology (KNUST), Kumasi, AK-385-1973 Ghana

**Keywords:** Genetic diversity, Genetic markers, Chickens, Population structure, SilicoDArTs, DArTseq SNPs

## Abstract

**Background:**

The application of biotechnologies which make use of genetic markers in chicken breeding is developing rapidly. Diversity Array Technology (DArT) is one of the current Genotyping-By-Sequencing techniques allowing the discovery of whole genome sequencing. In livestock, DArT has been applied in cattle, sheep, and horses. Currently, there is no study on the application of DArT markers in chickens. The aim was to study the effectiveness of DArTSeq markers in the genetic diversity and population structure of indigenous chickens (IC) and SASSO in the Eastern Province of Rwanda.

**Methods:**

In total 87 blood samples were randomly collected from 37 males and 40 females of indigenous chickens and 10 females of SASSO chickens purposively selected from 5 sites located in two districts of the Eastern Province of Rwanda. Genotyping by Sequencing (GBS) using DArTseq technology was employed. This involved the complexity reduction method through digestion of genomic DNA and ligation of barcoded adapters followed by PCR amplification of adapter-ligated fragments.

**Results:**

From 45,677 DArTseq SNPs and 25,444 SilicoDArTs generated, only 8,715 and 6,817 respectively remained for further analysis after quality control. The average call rates observed, 0.99 and 0.98 for DArTseq SNPs and SilicoDArTs respectively were quite similar. The polymorphic information content (PIC) from SilicoDArTs (0.33) was higher than that from DArTseq SNPs (0.22). DArTseq SNPs and SilicoDArTs had 34.4% and 34% of the loci respectively mapped on chromosome 1. DArTseq SNPs revealed distance averages of 0.17 and 0.15 within IC and SASSO chickens respectively while the respective averages observed with SilicoDArTs were 0.42 and 0.36. The average genetic distance between IC and SASSO chickens was moderate for SilicoDArTs (0.120) compared to that of DArTseq SNPs (0.048). The PCoA and population structure clustered the chicken samples into two subpopulations (1 and 2); 1 is composed of IC and 2 by SASSO chickens. An admixture was observed in subpopulation 2 with 12 chickens from subpopulation 1.

**Conclusions:**

The application of DArTseq markers have been proven to be effective and efficient for genetic relationship between IC and separated IC from exotic breed used which indicate their suitability in genomic studies. However, further studies using all chicken genetic resources available and large big sample sizes are required.

**Supplementary Information:**

The online version contains supplementary material available at 10.1186/s12864-024-10089-5.

## Background

Genomic selection using SNPs was developed so as to attain greater selection accuracy [1] and it offers another advantage of not depending solely on pedigree information [2]. In poultry breeding, genomic selection is, therefore, the new tool for genetic improvement allowing the simultaneous selection of multiple QTLs that affect a given quantitative trait. SNPs are the genotyping tools currently used in chickens, and are single nucleotide variants distributed across the DNA sequence [2]. SNPs provide an advantage in genotype calling precision [3].

The first 3 K SNP chip was developed in 2008 for 3,072 SNPs used for genotyping 2,576 DNAs isolated from both commercial layers and broilers [4]. The use of a limited number of breeds caused an allelic loss leading to a loss of genetic variability in commercial lines. Consequently, their use was limited. Three years later, a 60 K SNP chip was developed by [5]. The publication of this SNP chip was restricted to the public use [2, 6]. These authors reported another private 42 K SNP chip not publicly available developed by EW Group (Visbeck, Germany) comprising Lohmann Tierzucht (Cuxhaven, Germany), Aviagen (Huntsville, AL), and Hy-Line International (West Des Moines, IA). However, afterward, several publications from research works used this 42 K SNP chip [2]. So far, other SNP chips were developed including a 600 K SNP chip [7] with the advantage to be publicly available [6] and the recent 55 K SNP genotyping array developed from Chinese IC and some commercial chicken breeds [8]. Their characteristics to be bi-allelic as the most source of genetic variation, SNPs provide an advantage in genotype calling precision as reported by [9].

Diversity Array Technology (DArT) is one of the current Genotyping-By-Sequencing techniques allowing the discovery of whole genome sequencing [10]. Whole genome genotyping technique, sequence independence, ultra-high throughput, cost-effectiveness, and the huge number of markers over the whole genome [11] render DArT efficient in genomic studies. The markers from this technology offer the possibility of the analysis of population structure without prior information on the sequences [12, 13]. SilicoDArT, and Single Nucleotide Polymorphism (SNP), are two types of markers generated by DArT [ [14–17]. This technology has been widely applied in crops such as *Trema orientalis* a fodder crop [10], Cassava [18, 19], Garlic [20]. In livestock, DArT has been applied in cattle, sheep, and horses [21]. Currently, there is no study on the application of these markers in chickens. Only two studies have been reported on the genomic studies in indigenous chickens (IC) of Rwanda. These studies revealed high genetic variability among the Rwandan indigenous chickens with SSR [22] and SNPs [23] markers. In the first study SSR divided the Rwandan indigenous chickens into four gene pools (Central North and North West, Eastern, South West Central South, and the remaining chickens of South West). The second study dealt with the study of Genome-Wide Association (GWA) of the growth performance and antibody response (AbR) to Newcastle disease (ND) in Rwandan IC. Despite the efficiency of these markers used, their application are still a limitation since they are time consuming. However, genomic studies require reliable, rapid, and affordable costs for genotyping [6, 17].

In addition to this reason studies conducted so far are not sufficient for characterizing Rwandan IC and analyze their relationship with exotic breeds already available in the country. SASSO chickens are currently being introduced and spread at high level in the rural area across the country by Uzima Chick Ltd Company. However, SASSO is not a breed but chickens named after a French company name, SASSO (*Sélection Avicole* de la Sarthe et du *Sud Ouest* or Poultry Selection from Sarthe and South West). As explained by [24] and SASSO company on https://africa.SASSO-poultry.com/en/about-us/, the project was initiated by Serge Perrault in the 1950s with focus in preserving the traditional chickens in South France working on the SASSO T-line creation. After he collaborated with chicken farmers’ cooperative to create SASSO, a France-based poultry breeding firm with the purpose of developing the “Label Rouge” specifications from the French indigenous chickens. The aim of SASSO was to create red coloured birds under the “Label Rouge” certification standard. SASSO chicken were developed from French traditional chicken with focus in chicken types with abilities to produce eggs and/or meat in a range of invironment: free-range, backyard, or commercial while staying very productive and healthy. They were bred to be reared in a range of environments. They can be kept outdoors or indoor rearing systems. SASSO is currently part of Hendrix Genetics, a principal world-wide multi-species breeding corporation. Studies conducted by [24, 25] and [26] showed that SASSO chickens are characterised by grazing behaviour, a delicious and tender meat, and fast growth. This study was undertaken to analyze the effectiveness of Diversity Arrays Technology (DArT) as alternative markers in the study of the genetic diversity and population structure of IC and SASSO in the Eastern Province of Rwanda.

## Methods

### Study area

**Fig. 1:**
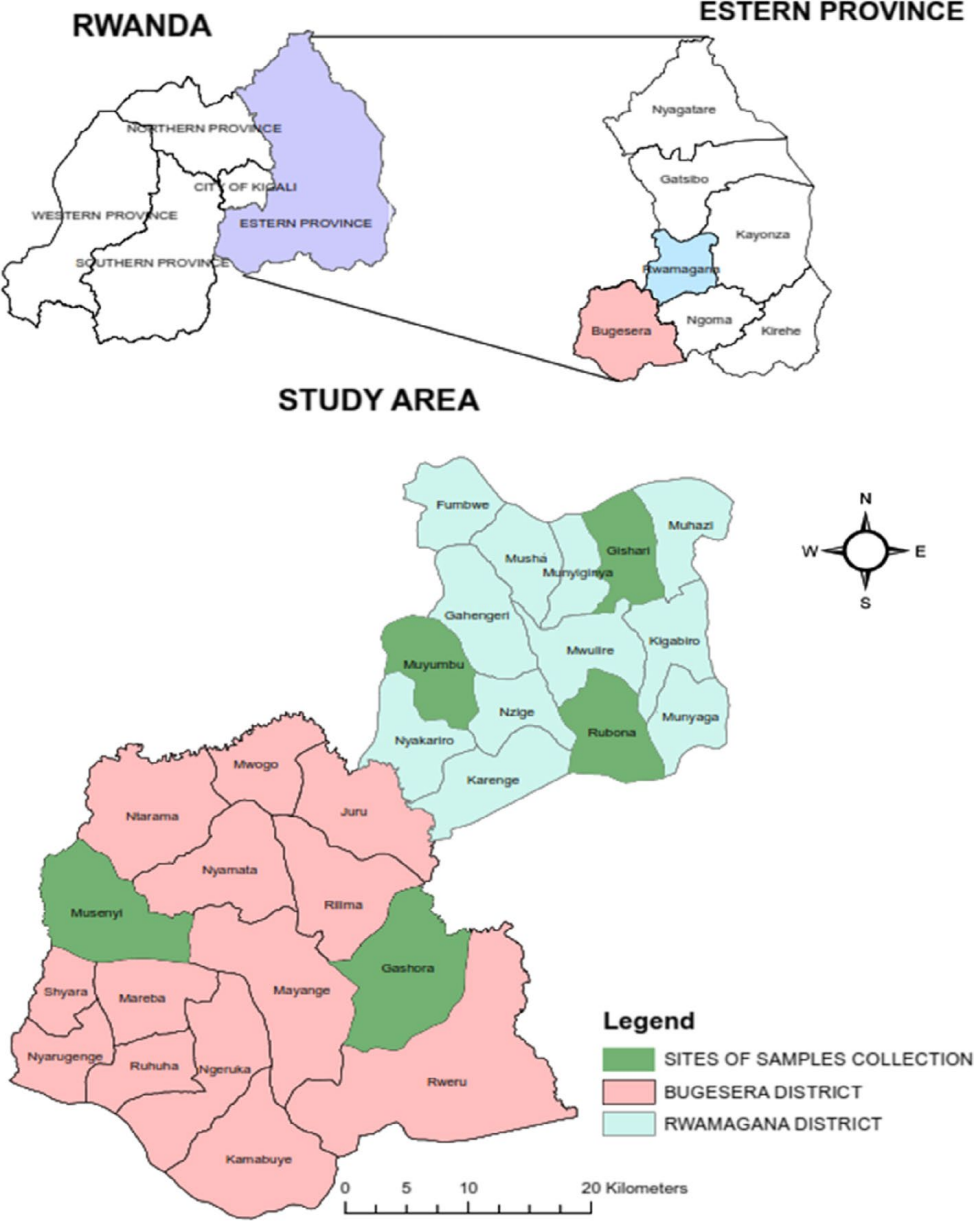
Sites of chicken blood samples collection

The study area covered the two sectors from Bugesera district and 3 sectors from Rwamagana district of the Eastern Province of Rwanda (Fig. 1). It was revealed that the gene flow in the Eastern Province was too low compared other gene pools identified [22]. In addition, Bugesera and Rwamagana districts which host a big number of IC border the City of Kigali considered as the target market of the chicken products (meat and eggs).

Two sectors in Bugesera districts included Musenyi and Gashora while three sectors from Rwamagana districts comprosed Gishari, Muyumbu and Rubona. Bugesera is located between 30^o^ 05’ East longitude, and 2^o^ 09’ South Latitude while Rwamagana lies between about 30° 26’ East Longitude and 1° 57’ 9” South Latitude. Ambient temperature ranged from 26^o^ C to 29° C and from 19^o^ C and 30^o^ C for Bugesera and Rwamagana respectively.

### Chicken blood sampling

Thirty-five [35] chicken blood samples were collected from Bugesera and 52 from Rwamagana districts (Fig. 1). Birds were purposively selected from indigenous and SASSO chickens from which individual blood samples were randomly collected. Thus, 77 samples from indigenous chickens (IC) and 10 blood samples SASSO chickens. The samples from Bugesera district were composed of 30 IC and 5 SASSO chickens while those from Rwamagana comprised 47 IC and 5 SASSO chickens (Table S1). SASSO chickens were collected from two private farms, one from each district. The samples were collected from chickens aged between 8 and 10 months for IC and 9 months for SASSO. IC were managed at rural households in extensive system with uncontrolled mating. Blood samples were collected from males and females IC. Fourteen males and 16 females were collected from sites in Bugesera while 23 males and 24 females were collected from sites in Rwamagana. The feeding was based on scavenging system with rare supplementation. SASSO chickens were commercial dual-purpose managed under intensive system and fed commercial layer diets. Blood samples were collected from SASSO females only. During sampling, to ensure that the sampled chickens were not genetically related, a distance between 500 and 800 m between households keeping chickens was considered referring to the sampling protocol in the study by [27]. With multi-drawing needles 21Gx1 1/2 (MED-833), after disinfection of the wing area, a professional and licensed Veterinarian collected 2 ml of blood from the wing vein in 4 ml-EDTA tubes. The tubes containing the blood samples were transported in a vaccine carrier box containing dry-ice. Then after, the samples were kept in the biology laboratory of the University of Rwanda, College of Science and Technology at 4^o^C. Within 24 h, the blood was subsampled in a sterilized area, into 1 µl each in 87 wells of a PCR 96-well plate using the pipette and pipette tips. The PCR 96-well plate was covered and sealed, then transported at ambient temperature and sent to the laboratory at International Livestock Research Institute (ILRI)-Nairobi in Kenya for genotyping services.

### DNA extraction and genotyping by sequencing

Chicken blood samples were sent to Integrated Genotyping Service and Support (IGSS) platform, currently changed to SEQART AFRICA located at Biosciences Eastern and Central Africa (BecA-ILRI) Hub in Nairobi for Genotyping. DNA extraction was done using the Nucleomag extraction kit. The genomic DNA extracted was in the range of 50-100ng/ul. DNA quality and quantity were checked on 0.8% agarose. Libraries were constructed according to [17] DArTSeq complexity reduction method through digestion of genomic DNA and ligation of barcoded adapters followed by PCR amplification of adapter-ligated fragments. Libraries were sequenced using Single Read sequencing runs for 77 bases. Next-generation sequencing was carried out using Hiseq2500. The IGSS platform uses a genotyping by Sequencing (GBS) DArTseq™ technology, which provides rapid, high quality, and affordable genome profiling, even from the most complex polyploid genomes. DArTseq markers scoring was achieved using DArTsoft14 which is an in-house marker scoring pipeline based on algorithms. Two types of DArTseq markers were scored, SilicoDArT and SNP markers which were both scored as binary for presence /absence (1 and 0, respectively) of the restriction fragment with the marker sequence in the genomic representation of the sample. Both SilicoDArT and DArt seq SNP markers were aligned to the reference genome of Chicken v5 to identify chromosomes and positions (Gallus_gallus-5.0 - Genome - Assembly - NCBI (nih.gov).

### Genetic diversity and population structure

 Before proceeding to further analyses, the markers were checked for their quality. Sex chromosomes for both DArTseq SNP and SilicoDArT marker data were removed as well as the markers without defined positions on chromosomes. The strict quality control of both DArTseq SNP and SilicoDArT markers, as well as samples, was performed by the package dartR 2.0.4 of R statistical software [28]. This package was furthermore used for data imputation to avoid the effect of missing values on the statistical analysis. The call rate, reproducibility, monomorphism, and One Ratio were the quality control parameters used. The call rate is explained as the percentage of samples for which the genotype call is either “1” or “0”. Reproducibility represents the percentage of technical replicate assay pairs for which the marker score is consistent, and monomorphic loci are those that represent one individual’s nucleotide state across individuals in a given population. One Ratio is the parameter explaining the percentage of samples for which the genotype score is “1”. The markers with a call rate ≥ 0.95, Reproducibility (RepAvg) of 1.0, One Ration > 0.05, and non-monomorphic were retained. Individuals with a call rate ≥ 0.90 were similarly retained for further analysis. The barplot function of R.4.0.5 statistical tool was used to plot the distribution of the alleles in both DArTseq maker datasets. Loci across the chicken genome were mapped using the KDCompute 1.5.2. beta, data analysis software (https://kdcompute.seqart.net/kdcompute/login) from Diversity Arrays Technology Pty Ltd. The Mantel test of the package “vegan” [29] of the R statistical tool was used to determine the correlation between DArTseq SNP and SilicoDArT marker systems where a non-parametric test with 10,000 random iterations was employed. The package “ggplot2” of the same program was employed to generate the scatterplot of the Mantel test. The genetic relationship among the chicken samples and origins was estimated using Nei’s 1972 genetic distance [30] for both sets of DArT marker systems. Nei’s 1972 genetic distance matrix and hierarchical dendrogram were generated using the “StAMPP” package [31] and “cluster” package [32] respectively both of the R statistical tool. The Principal Coordinate Analysis was determined using the package “dartR” of the R statistical program. STRUCTURE 2.3.4 software [33] was used to determine the population structure of chicken samples, where there was an independency between loci and admixture model, the Bayesian clustering method was used. Six replicate runs of 30,000 Markov Chain Monte Carlo (MCMC) iterations were performed after a burn-in period of 30,000 iterations of each cluster with k value ranging from 1 to 6. The number of clusters, k, was determined by the Evanno’s ∆k method of STRUCTURE Harvester vA.2 [34, 35].

## Results

### The quality and distribution of DArTseq SNP and SilicoDArT markers on the chicken genome

**Fig. 2:**
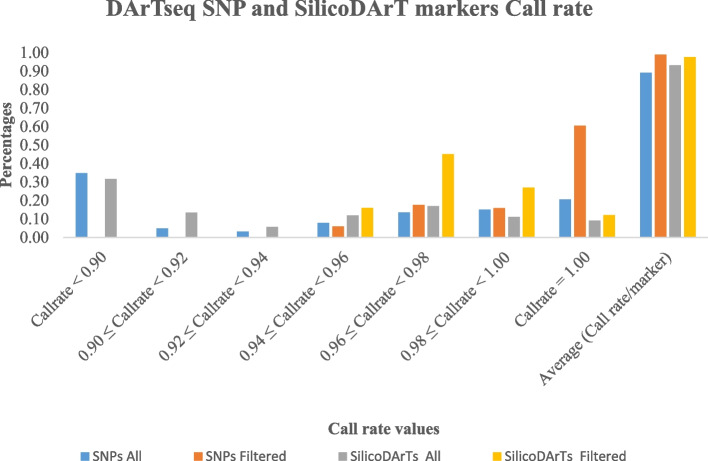
Call Rate of DArTseq SNP and SilicoDArT markers

Both datasets of DArTseq SNP (Table S2) and SilicoDArT (Table S3) markers and their metadata (Table S4) had undergone quality control before analysis. After removing the sex-linked loci and loci without positions on chromosomes, 40,832 DArTseq SNP and 20,920 SilicoDArT markers remained for the quality control process. This process left 8,715 DArTseq SNPs, 6,817 SilicoDArTs, and 86 samples for further analysis. The DArTseq SNP and SilicoDArT markers (Fig. 2) which showed less than 0.90 call rate before the quality scrutiny were 35% and 32% respectively. This study showed that 20% of SilicoDArTs had a call rate higher or equal to 0.98 including 9% which had a call rate of 1.0. The DArTseq SNPs which had a call of 0.98 were 36% comprising 21% with a call rate of 1.0. After quality scrutiny, 76% and 39% of DArTseq SNPs and SilicoDArTs respectively showed a call rate higher or equal to 0.98 (Fig. 2). The study showed some discrepancies in the quality of both markers based on the call rate; most of the DArTseq SNP markers (60%) scored a call rate of 1.0 while majority of SilicoDArT markers (45%) scored a call rate ranging from 0.96 to 0.98, with 12% scoring a call rate of 1.0. After the quality screening, a high number of SilicoDArT markers (72%) showed a call rate equal to or above 0.96 but less than 1 compared to DArTseq SNP markers (34%). The average call rate for DArTseq SNP and SilicoDArT markers were 0.89 and 0.93 respectively before the quality screening and 0.99 and 0.98 after.

**Fig. 3:**
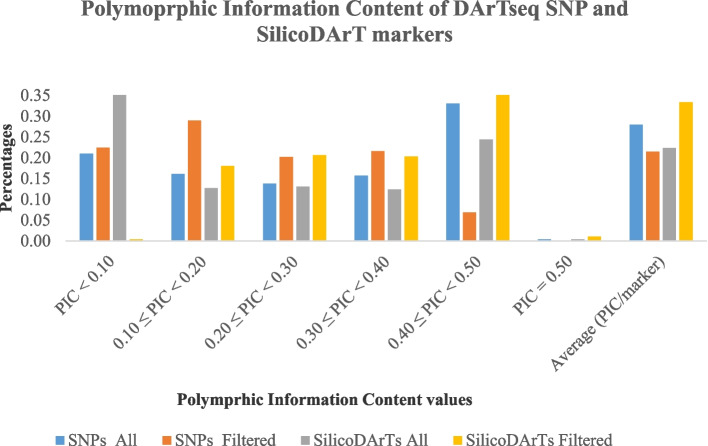
Polymorphic Information Content (PIC) of DArTseq SNP and SilicoDArT markers

**Fig. 4:**
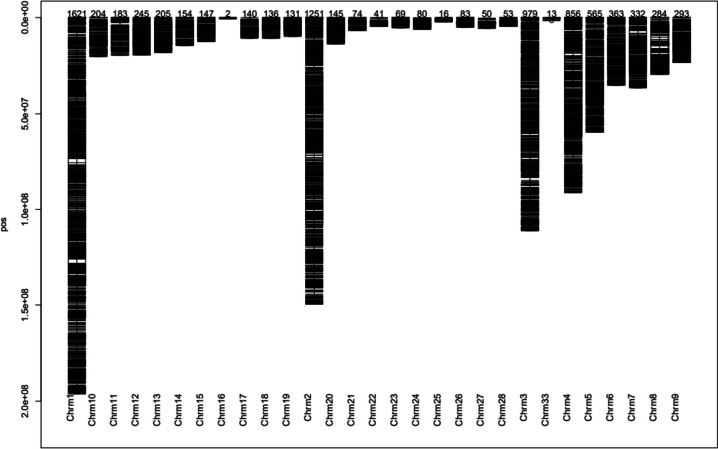
Distribution of DArTseq SNP markers on the chicken chromosomes

**Fig. 5:**
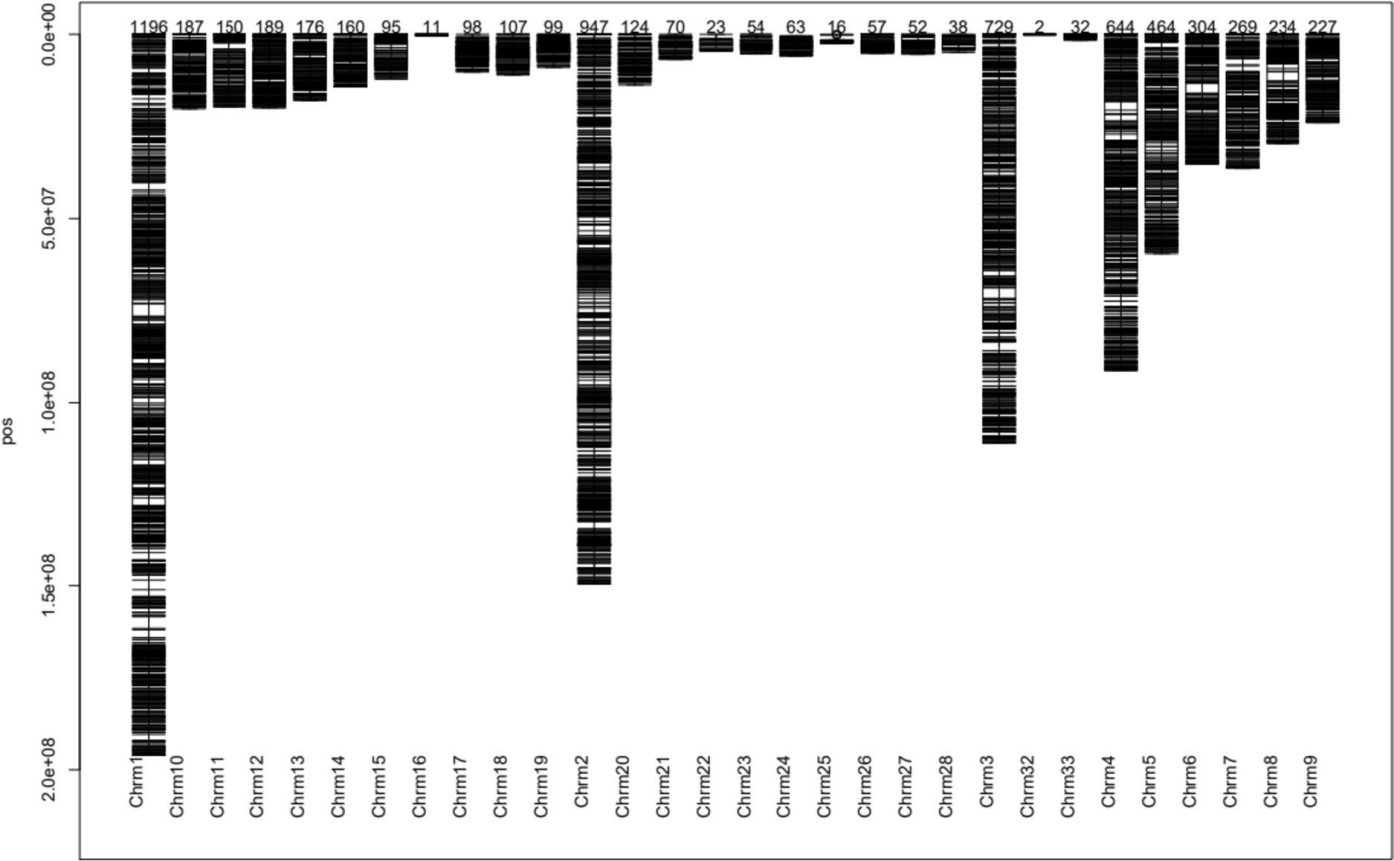
Distribution of SilicoDArT markers on the chicken chromosomes

The Polymorphic Information Content (PIC) less than 0.3 was shown by 51% and 63% of DArT seq SNP and SilicoDArT markers respectively before the quality scrutiny, and then 72% and 39% respectively after (Fig. 3). The number of markers increased by 21% for DArTseq SNPs and decreased by 24% for SilicoDArTs. The selected loci were mapped across the chicken genome (Figs. 4 and 5).

The results indicated that 8715 DArTseq SNP markers were mapped on 29 chromosomes (Fig. 4) while 6817 SilicoDArT markers were mapped on 30 chromosomes (Fig. 5). Two informative SilicoDArTs markers were mapped on chromosome 32 while there were no informative DArTseq SNP markers mapped on this chromosome. More than a half of DArTseq SNP markers (54%) and SilicoDArT markers (51.6%) were observed on the first four chromosomes comprising 34.4% (1621 DArTseq SNP markers) and 34% (1196 SilicoDArT markers) respectively mapped on chromosome 1. After loci mapping on autosomal chromosomes, the alternate alleles were counted in the function of loci across the genome.

**Fig. 6:**
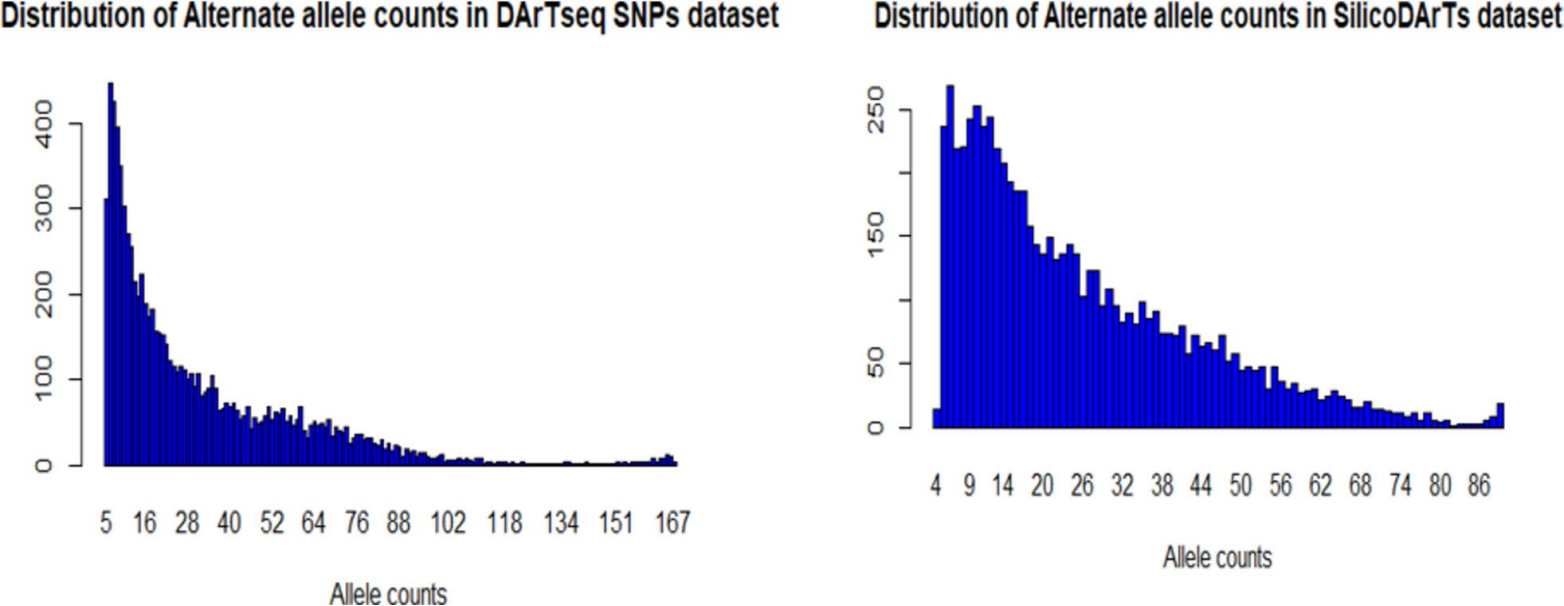
Distribution of ALT allele counts in DArTseq SNP (left) and SilicoDArT (right) datasets

The results revealed that the number of alternate alleles counts of DArTseq SNP markers was higher than that of SilicoDArT markers (Fig. 6). The number of alternate alleles counts varied from 5 to 167 for SNP markers and from 4 to 89 for SilicoDArT markers.

The respective average allele counts for DArTseq SNPs and SilicoDArTs were 31.7 and 25.7. Therefore, as the normality of both distributions was not tested and assuming that the averages of allele counts can be distorted by the high values in the datasets, the median can be a better to in describing data. As such, this study revealed that 21 allele counts were the median of both explanatory DArTseq SNP and SilicoDArT markers. Referring to the median, this study showed the same level of abundance of alternate alleles across the chicken genome in both datasets. The determination of allele counts over loci was followed by the analysis of the extent to which the mutation phenomena occurred across the genome of chicken samples used in this study. In the remaining DArTseq SNPs after quality check, as demonstrated in Table 1, the analysis of two types of DNA substitution mutations (transitions and transversions) showed that, transition SNPs (72.1%) were higher than the transversion SNPs (27.9%).

**Table 1:** Distribution of types of DNA substitution mutations across the chicken genome

Types of SNPs	Purines/Pyrimidines	No of allele sites	Frequency
**Transitions**	AG	3051	0.350
	CT	3233	0.371
	Total	6284	0.721
**Transversions**	AC	678	0.078
	AT	529	0.061
	GC	632	0.073
	GT	592	0.068
	Total	2431	0.279
**G/Total**		8715	1.000

*A *Adenine, *C *Cytosine, *G *Guanine, *T *Thymine

Among mutations revealed across the chicken genome (Table 1), C/T transitions showed to be frequent (37.1%) followed by the A/G transitions (35%) while the A/T transversions had the lowest frequency (6.1%) but similar to G/T transversions (6.8%).

### Genetic relationship between chicken samples

The genetic dissimilarities of the 86 chicken samples were analyzed using Nei’s distance matrix. The genetic dissimilarities based on DArTseq SNP markers varied from 0.09 to 0.22, with an average of 0.17 (Table S5). The chicken samples excluding SASSO samples revealed almost distance indices ranging from 0.09 (Muyumbu and Gishari) to 0.20 (Muyumbu and Musenyi) with a relatively similar average of 0.16–0.17. The distance indices for SASSO samples ranged from 0.09 to 0.17 with an average of 0.15 which is close to that of all IC samples of 0.17. Using SilicoDArT markers, the genetic distance indices varied between 0.22 and 0.51 with an average of 0.43 (Table S6). Similar to DArTseq SNP markers, the chicken samples from different origins showed a similar average of genetic dissimilarity of 0.42. Chicken samples from SASSO showed a slightly lower genetic dissimilarity ranging from 0.22 to 0.39 with an average of 0.36 compared to that of IC samples of 0.42. Comparing the two marker systems, both makers elucidated that the samples were distantly related but clearly by SilicoDArT markers than the DArTseq SNP markers. This was confirmed by Nei’s 1972 distance matrix (Table 2) showing the estimated dissimilarity indices of the origins of chicken samples. On one side, DArTseq SNP markers revealed a low dissimilarity between all genotype’s origins (0.010 to 0.056). The distance indices of IC origins varied between 0.010 and 0.030 with an average of 0.019 while those of SASSO and the origins of IC samples ranged from 0.040 to 0.056 with an average of 0.048. On the other hand,

**Table 2:** Nei’s 1972 genetic distance of origins of 86 chicken samples (SNP markers above the diagonal and SilicoDArT markers below diagonal

	Gishari	Musenyi	Gashora	Muyumbu	SASSO	Rubona
**Gishari**	0.000	0.013	0.019	0.010	0.042	0.024
**Musenyi**	0.037	0.000	0.017	0.011	0.049	0.025
**Gashora**	0.051	0.045	0.000	0.017	0.053	0.030
**Muyumbu**	0.028	0.030	0.045	0.000	0.040	0.022
**SASSO**	0.105	0.118	0.130	0.100	0.000	0.056
**Rubona**	0.066	0.069	0.082	0.063	0.147	0.000

**Fig. 7:**
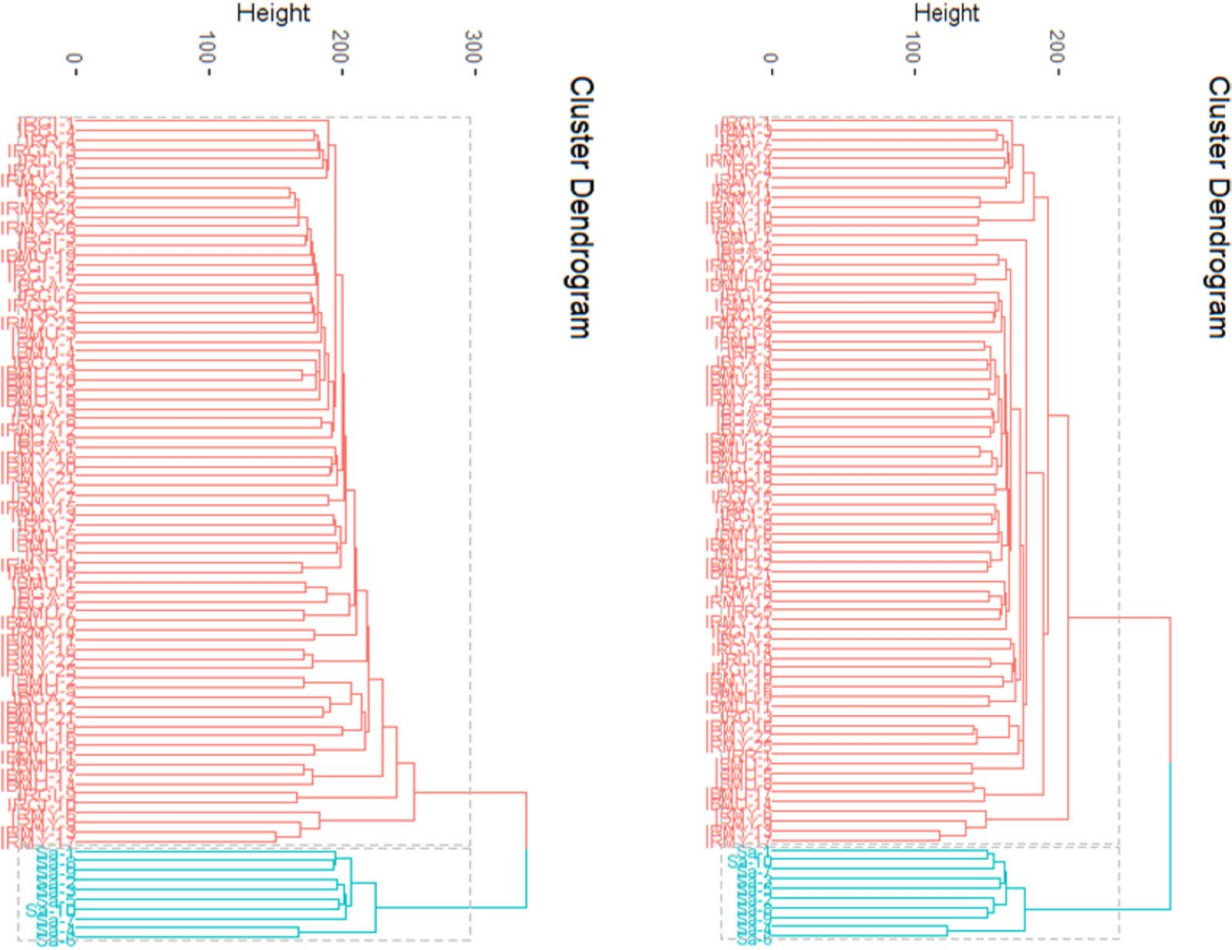
Dissimilarity dendrogram based on DArTseq SNP (left) and SilicoDArT (right) markers of chicken samples

SilicoDArT markers showed moderate dissimilarity between all samples ranging from 0.028 to 0.147. The dissimilarity indices between origins of IC samples were from 0.028 to 0.082 with an average of 0.056 while the distance indices between SASSO samples and origins of IC were from 0.100 to 0.147 with an average of 0.120. Nevertheless, using both marker systems, these results indicated that there is wide genetic distances among chicken samples but low genetic dissimilarities between IC origins and moderate genetic distance between IC origins and SASSO samples. Based on the genetic distances, the chicken samples formed two groups; the first group was composed of the IC samples from all origins, and the second comprised of SASSO samples. The genetic dissimilarity of the chicken samples was confirmed by the dendrogram produced by the Euclidian method of the package “cluster” of R statistical software (Fig. 7). Both marker systems grouped chicken samples into two separate clusters; one cluster was composed of IC from all origins (red color) and the other cluster was formed by SASSO chicken samples (blue color).

### The analysis of genetic diversity and population structure of chicken samples

**Fig. 8:**
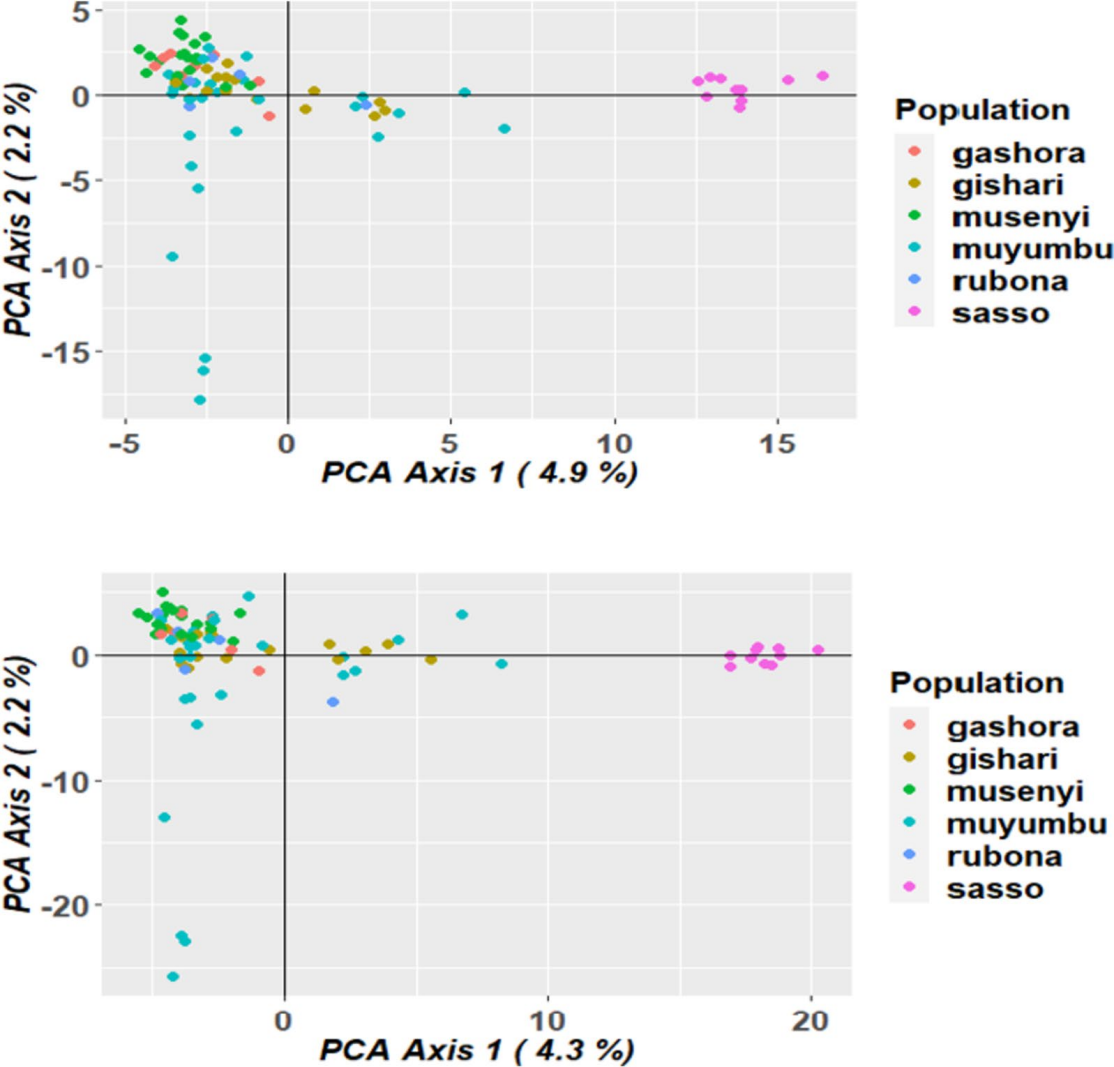
Principal Coordinate Analysis (PCoA) used to explain the genetic diversity across chicken samples based on DArTseq SNP (up) and SilicoDArT markers (down)

The Principal Coordinate Analysis (PCoA) was performed by dartR package based on both marker datasets to elucidate the genetic diversity among the 86 chicken samples and their origins. The principal components explained the low genetic variance for both marker categories. Considering the DArTseq SNP markers, the first two components explained the total variance of 7.1% (Fig. 8 up) while for SilicoDArT markers, the total variance explained by the first two components is 6.5% (Fig. 8 down).

**Fig. 9:**
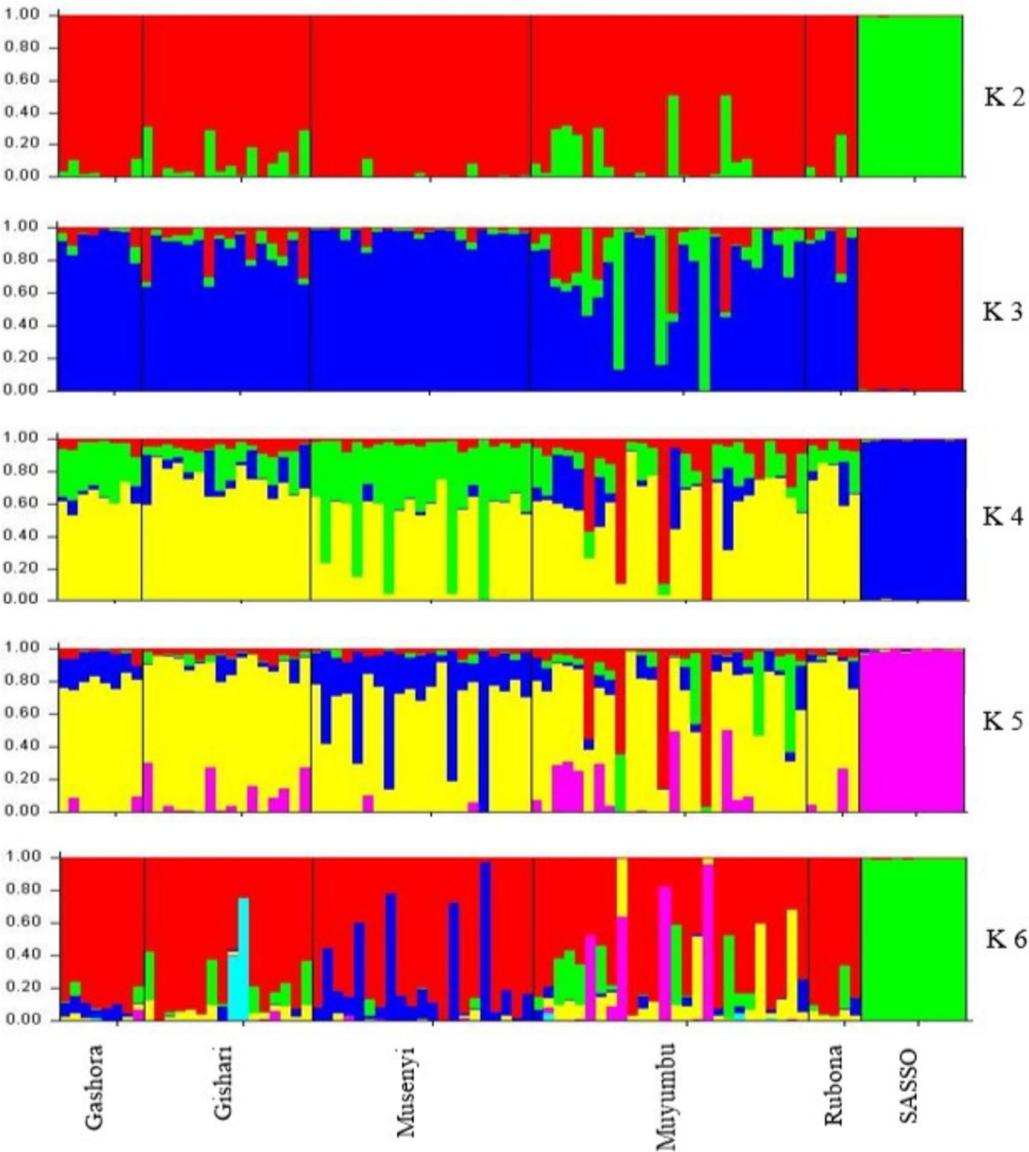
Population structure of chicken samples based on DArTseq SNP markers

**Fig. 10:**
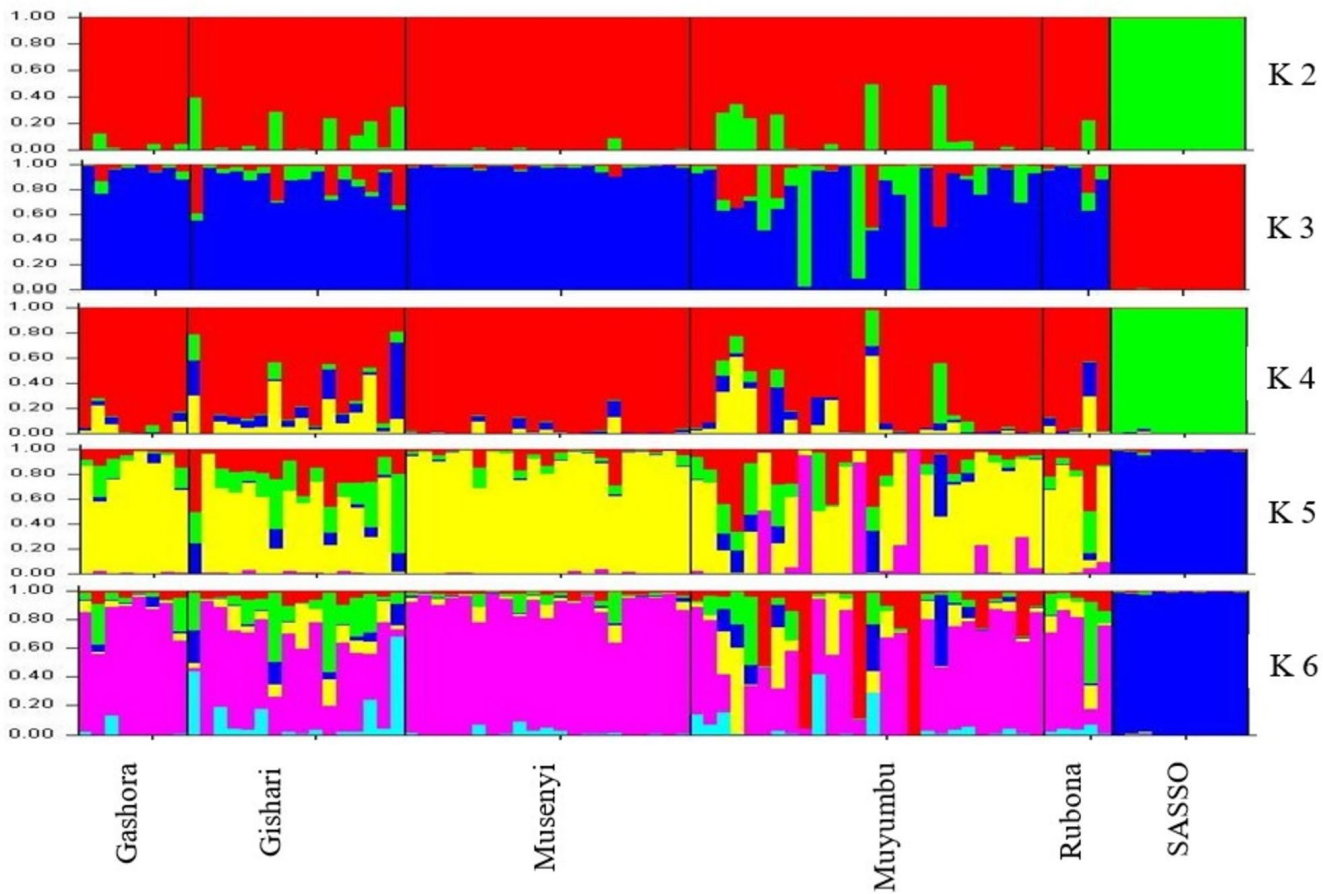
Population structure of chicken samples based on SilicoDArT markers

For both marker datasets, 86 chicken samples were subdivided into two groups similar to the hierarchical clusters where the SASSO samples were far from IC samples. IC samples from different regions clustered together apart from the SASSO samples, which clustered closely together. However, in both PCoA plots, 6 chickens from Muyumbu, 5 chickens from Gishari. and 1 chicken from Rubona had genetic relationship with SASSO chickens. Similarly, these plots showed that there was a low genetic diversity among the IC samples especially samples from Muyumbu and Gishari. The STRUCTURE program, using the Bayesian clustering model, was used in the analysis of the genetic structure of the chicken population based on both DArTseq markers (Figs. 9 and 10) and the population structure was similar.

The assignment of individual samples was similar for both DArT markers. From K 2 to K 6, SASSO Chickens were separated from IC and formed an isolated group. At K 6, the cluster 5 grouped 61 IC: all samples from Musenyi [21], 16 samples from Muyumbu, 12 from Gishari, 8 from Gashora, and 4 samples from Rubona. At K5, these samples were similarly grouped together at cluster 4 but with only 11 samples from Gishari. At K 4, cluster 1 grouped 71 samples together with 23 samples from Muyumbu, 21 from Musenyi, 14 from Gishari, all samples from Gashora and Rubona. At K 3, cluster similarly 3 grouped 71 samples; 21 samples from Muyumbu, and all samples from Gashora, Gishari, Musenyi, and Rubona.

The logarithm probability of ΔK = 190.4 for DArTseq SNP markers, and ΔK = 292.12 for SilicoDArT markers, which peaked at K = 2. This value of K means that chicken samples were clustered into two groups as the optimal number of subpopulations (Sub-pop A and Sub-pop B) and then, chicken individuals were assigned to their respective sub-populations (Table 3). Based on the DArTseq SNP marker dataset, 82.8% of chicken individuals were assigned to Sub-pop A while 17.2% were assigned to Sub-pop B. Referring to the SilicoDArT marker dataset, the STRUCTURE program assigned 82.9% of chicken samples in Sub-pop A and 17.1% in Sub-pop B. Sub-pops A and B were colored red and green respectively in both plots (Figs. 9 and 10). Sub-pop A comprised IC samples while Sub-pop B was composed of the SASSO chicken samples, confirming results revealed by the hierarchical clusters. The majority of IC samples representing 62.8% and 53.3% in SilicoDArT and DArTseq SNP datasets respectively were fully assigned to Sub-pop A, while all 10 SASSO chicken samples (100%) were fully assigned to Sub-pop B. An admixture of the genetic composition of Sub-pop A with Sub-pop B, characterized by ≥ 1.5% of ancestry from Sub-pop B, was observed. In total, 12 IC samples representing 15.8% of Sub-pop A showed to be in admixture with Sub-pop B, and these qualified as heterogeneous individuals.

The STRUCTURE estimated the average genetic diversity among the individual chicken samples within each subpopulation and between the two subpopulations expressed as the expected heterozygosity and Net nucleotide distance respectively (Table 3). The Net nucleotide distance between Sub-pop A and Sub-Pop B based on the DArTseq SNP markers was 0.021. The SilicoDArT markers revealed an estimate of a Net nucleotide distance of 0.008 between the two subpopulations indicating that they were widely related. The average genetic diversity among individuals (He) was 0.244 and 0.249 in Sub-pop A and Sub-pop B respectively in DArTseq SNP markers while the respective expected heterozygosity in both subpopulations was 0.234 and 0.247 based on SilicoDArT markers.

**Table 3:** Characteristics of clusters of 86 chicken samples

Net nucleotide distance		Membership probability	He
*DArTseq SNP markers*			
	**Sub-pop B**		
**Sub-pop A**	0.021	0.824	0.244
**Sub-pop B**		0.176	0.249
*SilicoDArT markers*			
	**Sub-pop B**		
**Sub-pop A**	0.008	0.829	0.234
**Sub-pop B**		0.171	0.247

These IC samples comprised 6 individuals from Muyumbu, 5 individuals from Gishari, and 1 individual from Rubona. The admixture explains the gene flow in the region of study, which confirmed what was observed in the principal coordinate analysis.

### Relationship between DArT SNP and SilicoDArT marker systems

**Fig. 11:**
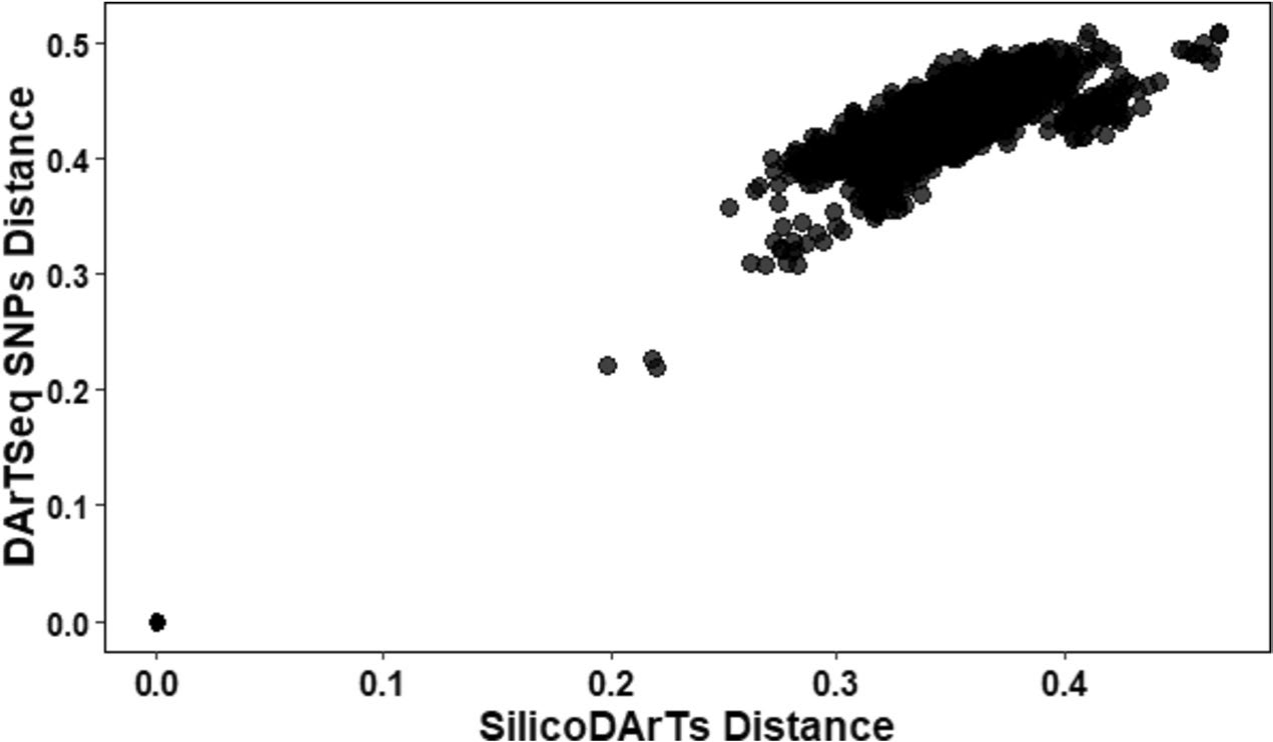
Correlation between SilicoDArT and DArTseq SNP marker datasets

The Mantel test applied to both DArT marker systems, based on Nei’s 1972 genetic distance matrices produced from DArTseq SNP and SilicoDArT markers illustrated a high relationship (*r* = 0.805; *p* < 0.001) between the two DArT marker systems (Fig. 11).

## Discussion

### The reliability and effectiveness of DArT marker platforms

A wide range of chicken breeds are kept under different conditions across the globe. A complex network drives the traits of economic importance between several genes across the genome [17]. Therefore, breeding programs conservation programs require the level of genome profiling of the varied chicken genetic resources and the identification of potential parents for future generations. This study applied DArT markers to the genome sequencing of chickens, where it was highlighted that these markers are suitable in chicken genomic studies. The change in markers’ quality was noted. There was an increase in call rate averages of 0.10 and 0.5 of DArTseqSNP and SilicoDArT markers respectively. The Polymorphic Information Content (PIC) less than 0.3 was shown by 51% and 63% of DArT seq SNP and SilicoDArT markers respectively before the quality scrutiny, then 72% and 39% respectively after. The percentage of markers increased by 21% for DArT seq SNPs while it decreased by 24% for SilicoDArTs. The low number of explanatory DArTseq SNP markers (7%) revealed to have a PIC higher or equal to 0.40 compared to 24.4% of SilicoDArT markers. These results explain the impact of the screening process on the markers’ quality. This discrepancy observed could be because the quality screening was performed on different quality components of markers. For example, all markers from DArT seq SNPs were screened based on both components One Ratio Reference and One Ratio SNPs while only SilicoDArTs were screened only on one component. In general, 100% of both marker systems were polymorphic after quality screening. The average of PIC for DArTseq SNP and SilicoDArT markers was 0.22 and 0.33 respectively. The Call rate average in this study presented by SNP markers was lower than that observed in the study of 55 K SNP genotyping array in different breeds in China [8] while the results of markers’ polymorphism were higher. In Chinese and exotic breeds, the averages of Call rates varied between 97% and 98.7% which is higher than 89% in or study, on other hand, 76.7% to 8% of SNPs were polymorphic, which is lower compared to 100% of SNPs observed in this study. A similar situation was observed with the samples’ assignment rate by DArTseq SNP markers observed in horses (96.7%), cattle (95.4%), and sheep (97%) in the study of Genotyping-by-sequencing performance in these species [21] using SNPs markers. The difference could be attributed to the quality screening process applied to these studies where the main criterion in the study in China was to discard the markers with minor Allele Frequency (MAF) ≥ 0.05 while addition to MAF > 0.01 in horses, cattle, and sheep, the markers were filtered based on default parameters set in the FastQC software, missing samples (*n* > 20% of individuals) and deviating from Hardy-Weinberg equilibrium with *p* < 0.001. However, these markers systems showed to be more polymorphic in chicken samples. In this study, DArTseq SNP markers were less informative than SilicoDArT markers after the strict quality control of the loci of both marker systems. This could mean that polymorphic SilicoDArT markers presented a large genome coverage than the DArTseq SNP markers. This difference in genome coverage and PIC of markers was observed in other studies that used DArTseq markers. A PIC between 0.40 and 0.50 was presented by 30% of SilicoDArT markers and 20% of DArTseq SNP markers in macadamia [36]. The study in cassava revealed the average of 0.28 and 0.36 for DArTseq SNP and SilicoDArT markers respectively [18], and respective PIC averages of 0.18 and 0.21 in Napier grass [12]. This was confirmed by their mapping on chicken chromosomes. The polymorphic markers of both systems were mapped differently on chicken chromosomes, where DArTseq SNP markers were mapped on 29 chromosomes and SilicoDArT markers on 30 chromosomes. It was revealed that there were no polymorphic DArTseq SNP markers mapped on chromosome 32 while only two SilicoDArT markers were observed on this chromosome. No polymorphic markers were mapped on chromosomes 29, 30, and 31 for both DArTseq SNP and SilicoDArT markers. Although both markers were abundantly mapped on four first chromosomes, the DArTseq SNPs showed to be more abundant (54%) than SilicoDArT markers (51.6%). Chromosome 1 was the chromosome that hosted a big number of both markers; 18.6% and 17.5% of DArTseq SNP and SilicoDArT markers respectively. Our results confirm the findings of the study on the diversity of *Napier grass* using the GBS method that the relative chromosomes are of different sizes [12]. Similar results of chromosome 1 to host a big number of markers were observed in horses (5.4%) and sheep (9.2%) in the study by [21] but the mapping rate in this study was higher. The Rwanda IC were genotyped with a low number of markers, 28 SSR [22] that could not cover the chicken genome. The location of markers could play an important role in GWA studies in which QTLs and candidate genes linked to markers can be identified. Therefore, the samples from different and varied regions of the entire genome render these high-density marker systems to reach a good genome coverage and be linked to a big number of candidate genes as there is a high correlation between the density of markers and that of genes [15, 17]. Mapping of both marker systems, evidencing their map positions on relative chromosomes is essential. Hence, this information could be necessary for the identification of the important regions on the genome which are able to control the economic traits in chickens based on the trait-marker association analysis. Compared to other genetic markers, the DArTseq marker systems have been revealed to be relevant in high-throughput work and provide the advantages of being time and cost-effective [17]. The loci mapping was enhanced by the determination of the alternate allele counts which were calculated by counting the number of any nucleotide bases, other than reference nucleotide bases found at a locus over the genome. Alternative alleles are referred to as single nucleotide variants [37]. The alternative allele counts were higher for DArTseq SNP markers than that for SilicoDarT markers. Assuming the distortion averages of both datasets due to high values and to the fact that they were not tested for the normality the median was found to be suitable for their comparison. It was revealed that there was no difference between the two dataset markers as the median of 21 alternative alleles per locus was similar. It means that both markers have a similar ability to be used for further genetic association studies between alternative allele counts and phenotypes for a given trait of economic importance [37, 38] in chickens. Some studies confirmed this association. The study by [38] tested the association between the ratio of alternative allele counts of the ASIP gene and Yoruba descent ancestry using linear regression to study the relationship between the genetic variability of this gene region and Yoruba ancestry. The authors observed the higher statistical power when the relationship between the genetic variability at a given region of the genome and phenotypes is tested by the association between alternative allele counts phenotypes. Similar results were observed in the study by [37] who tested the association between alternative allele counts (single nucleotide variants) of the BTBD18, a plausible candidate gene of childhood-onset obesity.

However, the alleles vary due to the extent to which the mutation phenomena occurred over a long period in population evolution. Two types of genetic mutations are frequent in population evolution; transitions and transversions. According to [39], are defined as transitions DNA mutations, when in the nucleotide base, the same number of rings is maintained with exchange from a purine to another purine (AG) or from pyrimidine to another pyrimidine (CT) while dissimilarly, transversions are genetic mutations that occur when the nucleotide base is changed from purine to a pyrimidine (AC; AT) or from pyrimidine to a purine (GC; GT). In our study, as demonstrated in Table 1, transition SNPs were higher than transversion SNPs. Among mutations revealed across the chicken genome, C/T transitions showed to be frequent followed by the A/G transitions while the A/T transversions had the lowest frequency but were closely similar to G/T transversions. It was revealed that the study influenced genetic variability may have on gene regulation had focused on primary cells and tissues in humans [39]. These authors, mentioned that knowing which types of mutations can most likely influence gene regulation could be a key to further investigation of their role in regulation variability in a population evolution. In their study, they concluded that the transversion mutations have a bigger impact than transition mutations on changes in protein synthesis due to the amino acid sequence alteration which results in changes in transcription factors (TFs) binding and phenotypes. Referring to our study’s results, gene regulation changes are likely to be low, which may indicate the high pressure on evolutionary selection on genes due to high transitions/transversions ratio equal to 2.6 [39]. As this is caused by the high depletion of transversions, the high pressure of selection on genes explains the low genetic variability in the chicken population observed at sites of study because the allele fixation increases as well (high rate of transitions). Similarly, to the conclusion of [39], the knowledge of types of mutations that occurred at a region of interest in the genome could be taken into consideration in genetics, especially for gene regulation for economic traits of interest that can assist in the breeding and conservation of our IC.

### Genetic relationship between chicken samples

In this study, the samples were collected from 5 different sites in the Eastern Province of Rwanda. The source of breeding stock in this area is mainly neighbor households and markets [22, 40]. Many chicken markets are found in various localities in the area so a close relationship between the chicken samples was expected. DarTseq SNP and SilicoDarT markers showed that the chickens’ samples were distantly similar. This study demonstrated that the distance indices from SilicoDArT markers were higher than those from DArTseq SNP markers. Results of genetic distance between IC origins in this study produced from DArTseq SNP (from 0.010 to 0.053) and SilicoDArT (from 0.028 to 0.082) markers were low compared to those found among 4 gene pools in the previous study in Rwanda (0.029 to 0.118) [22]. The genetic distances between exotic breeds and IC in this study were similarly lower to genetic dissimilarities in the previous study. The same differences were observed in the results from the study in Tanzania [27], in Iranian IC [41], in Chinese IC breeds and red jungle fowl [42], and in Ethiopian IC [43]. The results of this study were, nevertheless, higher than those observed in IC in Burkina Faso [44] for both DArT marker systems whilst they were low for SNPs markers and high for SilicoDArTs in three Chinese IC populations [45]. It, therefore, indicated that the SilicoDArTs could be more suitable for genetic diversity analysis in chickens than the DArTseq SNPs. The DArT markers used in this study in analyzing the genetic relationship of chicken samples grouped them into two groups; the first made up of IC chickens and the second composed of SASSO chickens. This was confirmed by the dendrograms which clustered all chicken samples into two clusters. The first cluster grouped IC from all origins while the second cluster grouped SASSO chickens only. Note that a cluster indicates the level of inbreeding that can characterize the populations sharing the same genetic material from a common identical ancestor [46].

### The analysis of genetic diversity and population structure of chicken samples

The Principal Coordinate Analysis (PCoA) for both marker systems subdivided the 86 chicken samples into two clusters similar to the hierarchical clusters where the SASSO samples are far from IC samples (Fig. 8). IC samples from different regions clustered together apart from SASSO samples that clustered alone, meaning that these two breeds do not share genetic material. However, a closer relationship between SASSO chickens and 12 IC comprising 6 chicken samples from Muyumbu, 5 chicken samples from Gishari, and 1 chicken genotype from Rubona assumed to be heterozygotes was noticed. Similarly, these plots showed that there is a low genetic diversity among the IC samples especially samples from Muyumbu and Gishari. These results were confirmed by the Bayesian clustering model performed by the STRUCTURE program used to analyze the structure of the chicken populations (Figs. 9 and 10). Similarly, IC samples from all origins clustered together forming group A separately from the SASSO samples clustered in group B. The degree of individual assignment of chicken samples to respective groups was similar for both marker systems. Similar population structure was observed in Tanzania between free-range local chickens [47] where two subpopulations were formed. Ching’wekwe and Morogoro-medium grouped together in one subpopulation while Kushi was isolated from them. This differentiation was enhanced by the the areas and climate conditions in which these ecotypes were reared and their origins. The coexistance of Ching’wekwe and Morogoro-medium in the same areas of similar climate conditions enhanced their similarity due to frequent interbreeding whileas Kush was separated from them because it is reared in humid and cooler zones. It was revealed that Kush and Shamo from Japan chare the mitochondrial DNA haplotypes suggesting its origin in Japan [48]. This confirms the admixture results observed in this study separating Rwandan IC from SASSO chickens having the origin in France.

The close relationship among IC chickens indicates the gene inflow in the sampling sites. The reproduction system in the area is based on the sharing of breeding stocks, especially the cocks. IC keepers either mate their hens to the cocks from their neighbors or buy cocks or hens from trans-bordering local markets. Another factor could be culture-based from birds’ exchange by donation. Note that when the samples show that they are closely related genetically, that means they share the same genetic material [49], especially through the mating systems in the sampling region. There was a correlation between genetic and geographic distances corroborating the study conducted previously in Rwanda by [22] and in Kenya [50]. Consequently, the geographic distances among the sampling sites influenced the population structure of 76 IC samples. In this study, an admixture of genetic composition among the two groups was observed. SASSO chickens were recently introduced in the rural area by various stakeholders in the poultry value chain especially NGOs and some hatcheries represented by UZIMA Chick Company Ltd to improve the livelihood of rural households referring to their adaptability to harsh environments like IC. These chickens have been spread across the Rwamagana district for few years. In the study site, some households keep either SASSO cocks, hens, or a mixture of hens and cocks. These birds are raised in the same systems as the IC, especially the scavenging system predominant in the area [40]. The mating in this system is random explaining the uncontrolled crossbreeding that occurred between IC and the SASSO chickens. It is worth noting that not only the random mating caused this gene flow in the region but also the intentional crossing made by the chicken keepers to genetically improve their local chickens. This was confirmed by [22] in their study where the IC in South West of the country was clustered with exotic breeds. However, this gene flow was not observed in the East may be because the exotic strains of chickens were not spread yet in the area and SASSO chickens were not included in the study at that moment. Although there was an admixture between IC and SASSO chickens, the two groups were characterized by a close relationship explained by the Net nucleotide distance performed by the STRUCTURE program. This distance was too low for both DArT seq marker systems, but a little higher for DArTseq SNPs explaining their suitability in breed differentiation. Net nucleotide distance observed in this study was in range with that observed in the F2 crossbreds between Iranian chickens and exotic broiler breed (from 0.0148 to 0.0888) in the Illumina 60 K chicken Beadchip-based study by [51] for SNPs but higher than the Net nucleotide distance observed in SilicoDArTs (0.008). Thus, this distance indicates the genetic resemblance of both chicken groups. Another interesting characteristic of these two groups was the similar degree of genetic variability within each of them as explained by the expected heterozygosity (Table 3). In genetic diversity, heterozygosity is a good indicator of population status. Its consideration is therefore required [22]. The same authors stated that the level of constancy of a given population is indicated by its mean heterozygosity whereas low heterozygosity shows a high genetic constancy of the population [52]. The genetic variability, characterized by the expected heterozygosity observed in this study, was lower than that observed in many studies in the region. It was close to that observed in cattle (Ho = 0.242), sheep (Ho = 0.239), and horse (Ho = 0.253) populations based on observed heterozygosity [21]. It varied from 0.644 to 0.668 in the study by [22] on the study of genetic diversity and population structure of IC in Rwanda using microsatellite markers. In the study conducted in Kenya by [53] on the genetic diversity of 8 indigenous chicken populations in Kenya using microsatellite markers, the genetic variability ranged between 0.688 and 0.754. Another microsatellite marker-based study in Kenya on indigenous chickens collected from eight different regions revealed the expected heterozygosity ranging from 0.351 to 0.434 [54]. In Tanzania, the genetic variability with expected heterozygosity varying from 0.910 to 0.937 with an average of 0.925 was observed across 10 indigenous chicken populations in a Major Histocompatibility Complex-linked microsatellite LEI0258 marker-based study [27]. The expected heterozygosity for both markers in this study were lower than that observed by Asadollahi et al. (2021) in their Illumina 60 K chicken Beadchip-based study (> 0.42).

However, the characteristics of SASSO chickens including their adaptability to difficult conditions such as scavenging as a feed source and resistance to diseases could confirm the genetic relationship of both breeds. For that, further studies are needed to clarify the genetic relationship within and between these two genetic resources on a large scale using the same genetic markers. The genetic variations within samples and between two subpopulations could be the opportunity of exploiting them for the IC conservation and breeding programs through sustainable crossbreeding programs. This breeding approach will surely increase the heterosis and genetic variability among chickens that will serve as future breeding materials. Although Rwandan IC is genetically related to SASSO chickens in the study area, they present unique genetic features allowing them to be adapted to various ecozones across the country and serve as a multipurpose source in rural households. A similar observation was made in a study conducted in Brazil that confirmed the uniqueness of Brazilian Creole chickens, despite their close genetic relationship with chicken breeds from other countries [55]. Thus, the development of IC production should result in keeping them in practically sustainable production systems [55]. In this study, the two marker systems showed quite similar results which confirms their effectiveness in studies of genetic variability in chickens. Their association analysis with the Mantel test (Fig. 11) disclosed that DArTseq SNP and SilicoDArT markers are quite closely correlated in the genetic relationship and population structure of the chickens, which confirms their consistency. The consistency found here confirmed what was revealed in the study on cassava in Ghana [18]. The use of these markers could be extended to the analysis of genetic diversity and relationships within and between various chicken populations and ecotypes available in the site of study in particular and on a large scale in general.

## Conclusions

DArT markers, in this study, showed non-coverage on all chicken chromosomes and the criteria used for the quality control resulted into the loss of markers which require consideration in further investigations. The IC from the sites studied were genetically closely related and within each site they showed a moderate genetic variability. The relationship between SASSO chickens and IC was characterized by an admixture. Consequently, future breeding programs for good genetic improvement and conservation of IC could take into account other genetic resources available in the region and their relationship with them. In this study, both DarTseq SNPs and SilicoDArTs presented similar results confirming their effectiveness in the analysis of genetic diversity and population structure in chickens. Nevertheless, further studies to investigate or confirm the discrepancy observed between genetic markers are required when the sample size and the number of chicken populations are taken into consideration. The application of DArTseq markers have been proven to be effective and efficient for genetic relationship between IC and separated them from exotic breed used which indicate their suitability in genomic studies.

### Supplementary Information

**Additional file 1: Table S1. **Chicken individuals used in this study with their origins

**Additional file 2.**

**Additional file 3.**

**Additional file 4.**

**Additional file 5.**

**Additional file 6**

## Data Availability

The datasets generated and/or analysed during the current study are available in the OSF repository: https://osf.io/rtvp4/?view_only=a3be05a70a514b7fa20b17bd0bb982b1.

## Abbreviations

DArT: Diversity Array Technology
IC: Indigenous chickens
GBS: Genotyping by Sequencing
DNA: Deoxyribonucleic acid
PCR: Polymerase Chain Reaction
SNP: Single Nucleotide Polymorphism
PCoA: Principal Coordinate Analysis
QTLs: Quantitative Trait Loci
EDTA: Ethylenediamine tetra acetic acid
DRI: Directorate of Research and Innovation
ILRI: International Livestock Research Institute
PIC: Polymorphic Information Content
ASIP: Agouti-signaling protein
