# Supplementary material for: Effectiveness of DArTseq markers application in genetic diversity and population structure of indigenous chickens in Eastern Province of Rwanda
Source: BMC Genomics. 2024 Feb 19;25:193. doi: 10.1186/s12864-024-10089-5 (PMC10875757; doi:10.1186/s12864-024-10089-5)
Supplement: Supplementary file 1 — Additional file 1: Table S1. Chicken individuals used in this study with their origins [file 12864_2024_10089_MOESM1_ESM.docx]

**Table S 1 Chicken individuals used in this study with their origins**

| **ID** | **Site** | **District** | **ID** | **Site** | **District** | **ID** | **Site** | **District** |
| --- | --- | --- | --- | --- | --- | --- | --- | --- |
| rw1 | Gishari | Rwamagana | sa4 | Muyumbu | Rwamagana | rw34 | Muyumbu | Rwamagana |
| bu1 | Musenyi | Bugesera | bu10 | Musenyi | Bugesera | rw35 | Muyumbu | Rwamagana |
| bu2 | Gashora | Bugesera | bu11 | Musenyi | Bugesera | rw36 | Muyumbu | Rwamagana |
| rw2 | Gishari | Rwamagana | rw19 | Muyumbu | Rwamagana | sa7 | Gishari | Rwamagana |
| rw3 | Gishari | Rwamagana | bu12 | Musenyi | Bugesera | bu22 | Musenyi | Bugesera |
| rw4 | Muyumbu | Rwamagana | bu13 | Gashora | Rwamagana | bu23 | Musenyi | Bugesera |
| rw5 | Muyumbu | Rwamagana | rw20 | Gishari | Rwamagana | rw37 | Gishari | Rwamagana |
| rw6 | Muyumbu | Rwamagana | rw21 | Rubona | Rwamagana | bu24 | Gashora | Bugesera |
| sa1 | Muyumbu | Rwamagana | rw22 | Muyumbu | Rwamagana | rw38 | Gishari | Rwamagana |
| sa2 | Rubona | Rwamagana | rw23 | Muyumbu | Rwamagana | rw39 | Rubona | Rwamagana |
| bu3 | Musenyi | Bugesera | rw24 | Muyumbu | Rwamagana | rw40 | Muyumbu | Rwamagana |
| rw7 | Gishari | Rwamagana | sa5 | Musenyi | Bugesera | rw41 | Muyumbu | Rwamagana |
| bu4 | Musenyi | Bugesera | bu14 | Musenyi | Bugesera | rw42 | Muyumbu | Rwamagana |
| bu5 | Gashora | Bugesera | bu15 | Musenyi | Bugesera | sa8 | Gashora | Bugesera |
| rw8 | Gishari | Rwamagana | rw25 | Muyumbu | Bugesera | bu25 | Musenyi | Bugesera |
| rw9 | Gishari | Rwamagana | bu16 | Musenyi | Bugesera | bu26 | Musenyi | Bugesera |
| rw10 | Muyumbu | Rwamagana | bu17 | Gashora | Bugesera | rw43 | Gishari | Rwamagana |
| rw11 | Muyumbu | Rwamagana | rw26 | Gishari | Rwamagana | bu27 | Gashora | Bugesera |
| rw12 | Muyumbu | Rwamagana | rw27 | Rubona | Rwamagana | bu28 | Musenyi | Bugesera |
| sa3 | Gishari | Rwamagana | rw28 | Muyumbu | Rwamagana | rw44 | Gishari | Rwamagana |
| bu6 | Musenyi | Bugesera | rw29 | Muyumbu | Rwamagana | rw45 | Muyumbu | Rwamagana |
| bu7 | Musenyi | Bugesera | rw30 | Muyumbu | Rwamagana | rw46 | Muyumbu | Rwamagana |
| rw13 | Gishari | Rwamagana | sa6 | Gashora | Bugesera | rw47 | Muyumbu | Rwamagana |
| bu8 | Musenyi | Bugesera | bu18 | Musenyi | Bugesera | sa9 | Musenyi | Bugesera |
| bu9 | Gashora | Bugesera | bu19 | Musenyi | Bugesera | sa10 | Gashora | Bugesera |
| rw14 | Gishari | Rwamagana | rw31 | Gishari | Rwamagana | bu29 | Musenyi | Bugesera |
| rw15 | Rubona | Rwamagana | bu20 | Musenyi | Bugesera | bu30 | Musenyi | Bugesera |
| rw16 | Muyumbu | Rwamagana | bu21 | Gashora | Bugesera |  |  |  |
| rw17 | Muyumbu | Rwamagana | rw32 | Gishari | Rwamagana |  |  |  |
| rw18 | Muyumbu | Rwamagana | rw33 | Rubona | Rwamagana |  |  |  |
